# Correction: Molecular epidemiology of scrub typhus in Taiwan during 2006–2016

**DOI:** 10.1371/journal.pntd.0011025

**Published:** 2022-12-30

**Authors:** Hsiang-Fei Chen, Shih-Huan Peng, Kun-Hsien Tsai, Cheng-Fen Yang, Mei-Chun Chang, Yeou-Lin Hsueh, Chien-Ling Su, Ruo-Yu Wang, Pei-Yun Shu, Su-Lin Yang

There are errors in the Funding statement. It should read as follows: This work was supported by grants MOHW105-CDC-C-315-122110(PYS) and MOHW106-CDC-C-315-113112(PYS) from Centers for Disease Control, Ministry of Health and Welfare, Taiwan, Republic of China. The funder had no role in study design, data collection and analysis, decision to publish, or preparation of the manuscript.
