# Supplementary material for: Molecular epidemiology of scrub typhus in Taiwan during 2006–2016
Source: PLoS Negl Trop Dis. 2022 Apr 29;16(4):e0010369. doi: 10.1371/journal.pntd.0010369 (PMC9094550; doi:10.1371/journal.pntd.0010369)
Supplement: S1 Table — (PDF) [file pntd.0010369.s001.pdf]

No.	Isolated identifier	Strain name	Sequence type	length	Accession no
1	OT/Taiwan/KM0605a/2006	KM0605a	TW-1	1608	GQ332742
2	OT/Taiwan/KM0606b/2006	KM0606b	TW-1	1608	MW495302
3	OT/Taiwan/TY0606a/2006	TY0606a	TW-1	1608	MW495303
4	OT/Taiwan/KM0606c/2006	KM0606c	TW-1	1608	MW495304
5	OT/Taiwan/KM0607a/2006	KM0607a	TW-1	1608	MW495305
6	OT/Taiwan/KM0607c/2006	KM0607c	TW-1	1608	MW495306
7	OT/Taiwan/KM0607d/2006	KM0607d	TW-1	1608	MW495307
8	OT/Taiwan/KM0607e/2006	KM0607e	TW-1	1608	MW495308
9	OT/Taiwan/KM0607f/2006	KM0607f	TW-1	1608	MW495309
10	OT/Taiwan/KM0607g/2006	KM0607g	TW-1	1608	MW495310
11	OT/Taiwan/KM0608a/2006	KM0608a	TW-1	1608	MW495311
12	OT/Taiwan/KM0608b/2006	KM0608b	TW-1	1608	MW495312
13	OT/Taiwan/KM0608c/2006	KM0608c	TW-1	1608	MW495313
14	OT/Taiwan/TY0609a/2006	TY0609a	TW-1	1608	MW495314
15	OT/Taiwan/KM0705a/2007	KM0705a	TW-1	1608	MW495315
16	OT/Taiwan/KM0705c/2007	KM0705c	TW-1	1608	MW495316
17	OT/Taiwan/KM0706b/2007	KM0706b	TW-1	1608	MW495317
18	OT/Taiwan/KM0706c/2007	KM0706c	TW-1	1608	MW495318
19	OT/Taiwan/KM0706d/2007	KM0706d	TW-1	1608	MW495319
20	OT/Taiwan/KM0706e/2007	KM0706e	TW-1	1608	MW495320
21	OT/Taiwan/KH0706a/2007	KH0706a	TW-1	1608	MW495321
22	OT/Taiwan/KM0707b/2007	KM0707b	TW-1	1608	MW495322
23	OT/Taiwan/KM0707c/2007	KM0707c	TW-1	1608	MW495323
24	OT/Taiwan/KM0707d/2007	KM0707d	TW-1	1608	MW495324
25	OT/Taiwan/KM0707a/2007	KM0707a	TW-1	1608	MW495325
26	OT/Taiwan/KM0707h/2007	KM0707h	TW-1	1608	MW495326
27	OT/Taiwan/KH0707a/2007	KH0707a	TW-1	1608	MW495327
28	OT/Taiwan/KM0707i/2007	KM0707i	TW-1	1608	MW495328
29	OT/Taiwan/PT0704a/2007	PT0704a	TW-1	1608	MW495329
30	OT/Taiwan/TT0707a/2007	TT0707a	TW-1	1608	MW495330
31	OT/Taiwan/TCC0705a/2007	TCC0705a	TW-1	1608	MW495331
32	OT/Taiwan/LC0708a/2007	LC0708a	TW-1	1608	MW495332
33	OT/Taiwan/PT0709a/2007	PT0709a	TW-1	1608	MW495333
34	OT/Taiwan/PH0711a/2007	PH0711a	TW-1	1608	MW495334
35	OT/Taiwan/PH0711d/2007	PH0711d	TW-1	1608	MW495335
36	OT/Taiwan/PH0711e/2007	PH0711e	TW-1	1608	MW495336

37	OT/Taiwan/PT0712a/2007	PT0712a	TW-1	1608	MW495337
38	OT/Taiwan/KM0710d/2007	KM0710d	TW-1	1608	MW495338
39	OT/Taiwan/KM0710c/2007	KM0710c	TW-1	1608	MW495339
40	OT/Taiwan/KM0805b/2008	KM0805b	TW-1	1608	MW495340
41	OT/Taiwan/KM0806a/2008	KM0806a	TW-1	1608	MW495341
42	OT/Taiwan/KM0806b/2008	KM0806b	TW-1	1608	MW495342
43	OT/Taiwan/KM0806f/2008	KM0806f	TW-1	1608	MW495343
44	OT/Taiwan/KM0807a/2008	KM0807a	TW-1	1608	MW495344
45	OT/Taiwan/KM0807b/2008	KM0807b	TW-1	1608	MW495345
46	OT/Taiwan/KM0806g/2008	KM0806g	TW-1	1608	MW495346
47	OT/Taiwan/KM0807c/2008	KM0807c	TW-1	1608	MW495347
48	OT/Taiwan/KM0807d/2008	KM0807d	TW-1	1608	MW495348
49	OT/Taiwan/KM0807e/2008	KM0807e	TW-1	1608	MW495349
50	OT/Taiwan/KM0807j/2008	KM0807j	TW-1	1608	MW495350
51	OT/Taiwan/ML0807a/2008	ML0807a	TW-1	1608	MW495351
52	OT/Taiwan/KM0809b/2008	KM0809b	TW-1	1608	MW495352
53	OT/Taiwan/KM0809c/2008	KM0809c	TW-1	1608	MW495353
54	OT/Taiwan/KM0809d/2008	KM0809d	TW-1	1608	MW495354
55	OT/Taiwan/KM0810b/2008	KM0810b	TW-1	1608	MW495355
56	OT/Taiwan/KH0804a/2008	KH0804a	TW-1	1608	MW495356
57	OT/Taiwan/PH0805a/2008	PH0805a	TW-1	1608	MW495357
58	OT/Taiwan/KM0807f/2008	KM0807f	TW-1	1608	MW495358
59	OT/Taiwan/LC0807a/2008	LC0807a	TW-1	1608	MW495359
60	OT/Taiwan/LC0807b/2008	LC0807b	TW-1	1608	MW495360
61	OT/Taiwan/LC0807c/2008	LC0807c	TW-1	1608	MW495361
62	OT/Taiwan/LC0807e/2008	LC0807e	TW-1	1608	MW495362
63	OT/Taiwan/LC0807f/2008	LC0807f	TW-1	1608	MW495363
64	OT/Taiwan/LC0807g/2008	LC0807g	TW-1	1608	MW495364
65	OT/Taiwan/LC0807h/2008	LC0807h	TW-1	1608	MW495365
66	OT/Taiwan/TT0808a/2008	TT0808a	TW-1	1608	MW495366
67	OT/Taiwan/TT0807c/2008	TT0807c	TW-1	1608	MW495367
68	OT/Taiwan/LC0808b/2008	LC0808b	TW-1	1608	MW495368
69	OT/Taiwan/TC0809a/2008	TC0809a	TW-1	1608	MW495369
70	OT/Taiwan/KM0907j/2009	KM0907j	TW-1	1608	MW495370
71	OT/Taiwan/TP0909a/2009	TP0909a	TW-1	1608	MW495371
72	OT/Taiwan/KM0909a/2009	KM0909a	TW-1	1608	MW495372
73	OT/Taiwan/KM0908b/2009	KM0908b	TW-1	1608	MW495373

74	OT/Taiwan/KM0908a/2009	KM0908a	TW-1	1608	MW495374
75	OT/Taiwan/KM0907i/2009	KM0907i	TW-1	1608	MW495375
76	OT/Taiwan/KM0906c/2009	KM0906c	TW-1	1608	MW495376
77	OT/Taiwan/KM0907e/2009	KM0907e	TW-1	1608	MW495377
78	OT/Taiwan/KM0906b/2009	KM0906b	TW-1	1608	MW495378
79	OT/Taiwan/KM0907b/2009	KM0907b	TW-1	1608	MW495379
80	OT/Taiwan/KM0907c/2009	KM0907c	TW-1	1608	MW495380
81	OT/Taiwan/KM0907d/2009	KM0907d	TW-1	1608	MW495381
82	OT/Taiwan/HL0904a/2009	HL0904a	TW-1	1608	MW495382
83	OT/Taiwan/KH0904a/2009	KH0904a	TW-1	1608	MW495383
84	OT/Taiwan/PH0905a/2009	PH0905a	TW-1	1608	MW495384
85	OT/Taiwan/LC0811a/2008	LC0811a	TW-1	1608	MW495385
86	OT/Taiwan/PH0811a/2008	PH0811a	TW-1	1608	MW495386
87	OT/Taiwan/HL0906a/2009	HL0906a	TW-1	1608	MW495387
88	OT/Taiwan/LC0907a/2009	LC0907a	TW-1	1608	MW495388
89	OT/Taiwan/TP0906a/2009	TP0906a	TW-1	1608	MW495389
90	OT/Taiwan/LC0907b/2009	LC0907b	TW-1	1608	MW495390
91	OT/Taiwan/TT0907a/2009	TT0907a	TW-1	1608	MW495391
92	OT/Taiwan/LC0907c/2009	LC0907c	TW-1	1608	MW495392
93	OT/Taiwan/HL0909a/2009	HL0909a	TW-1	1608	MW495393
94	OT/Taiwan/HL0910a/2009	HL0910a	TW-1	1608	MW495394
95	OT/Taiwan/LC0911a/2009	LC0911a	TW-1	1608	MW495395
96	OT/Taiwan/TT1005a/2010	TT1005a	TW-1	1608	MW495396
97	OT/Taiwan/TNC1005a/2010	TNC1005a	TW-1	1608	MW495397
98	OT/Taiwan/KHC1006a/2010	KHC1006a	TW-1	1608	MW495398
99	OT/Taiwan/TPC1007a/2010	TPC1007a	TW-1	1608	MW495399
100	OT/Taiwan/LC1007a/2010	LC1007a	TW-1	1608	MW495400
101	OT/Taiwan/TT1009b/2010	TT1009b	TW-1	1608	MW495401
102	OT/Taiwan/TT1010a/2010	TT1010a	TW-1	1608	MW495402
103	OT/Taiwan/TT1004c/2010	TT1004c	TW-1	1608	MW495403
104	OT/Taiwan/KM1011a/2010	KM1011a	TW-1	1608	MW495404
105	OT/Taiwan/KM1010a/2010	KM1010a	TW-1	1608	MW495405
106	OT/Taiwan/KM1007d/2010	KM1007d	TW-1	1608	MW495406
107	OT/Taiwan/KM1007c/2010	KM1007c	TW-1	1608	MW495407
108	OT/Taiwan/HL1006b/2010	HL1006b	TW-1	1608	MW495408
109	OT/Taiwan/KM1006b/2010	KM1006b	TW-1	1608	MW495409
110	OT/Taiwan/KM1006a/2010	KM1006a	TW-1	1608	MW495410

111	OT/Taiwan/LC1106a/2011	LC1106a	TW-1	1608	MW495411
112	OT/Taiwan/TT1107a/2011	TT1107a	TW-1	1608	MW495412
113	OT/Taiwan/TT1108a/2011	TT1108a	TW-1	1608	MW495413
114	OT/Taiwan/KHC1107a/2011	KHC1107a	TW-1	1608	MW495414
115	OT/Taiwan/KM1107b/2011	KM1107b	TW-1	1608	MW495415
116	OT/Taiwan/KM1106e/2011	KM1106e	TW-1	1608	MW495416
117	OT/Taiwan/KM1107a/2011	KM1107a	TW-1	1608	MW495417
118	OT/Taiwan/KM1106d/2011	KM1106d	TW-1	1608	MW495418
119	OT/Taiwan/KM1106c/2011	KM1106c	TW-1	1608	MW495419
120	OT/Taiwan/KM1106b/2011	KM1106b	TW-1	1608	MW495420
121	OT/Taiwan/KM1106a/2011	KM1106a	TW-1	1608	MW495421
122	OT/Taiwan/LC1207a/2012	LC1207a	TW-1	1608	MW495422
123	OT/Taiwan/LC1208a/2012	LC1208a	TW-1	1608	MW495423
124	OT/Taiwan/LC1208b/2012	LC1208b	TW-1	1608	MW495424
125	OT/Taiwan/TT1208a/2012	TT1208a	TW-1	1608	MW495425
126	OT/Taiwan/TT1209a/2012	TT1209a	TW-1	1608	MW495426
127	OT/Taiwan/LC1211a/2012	LC1211a	TW-1	1608	MW495427
128	OT/Taiwan/TT1212c/2012	TT1212c	TW-1	1608	MW495428
129	OT/Taiwan/KM1205a/2012	KM1205a	TW-1	1608	MW495429
130	OT/Taiwan/KM1206d/2012	KM1206d	TW-1	1608	MW495430
131	OT/Taiwan/KM1305a/2013	KM1305a	TW-1	1608	MW495431
132	OT/Taiwan/KM1306a/2013	KM1306a	TW-1	1608	MW495432
133	OT/Taiwan/KM1306b/2013	KM1306b	TW-1	1608	MW495433
134	OT/Taiwan/KM1306c/2013	KM1306c	TW-1	1608	MW495434
135	OT/Taiwan/KM1307a/2013	KM1307a	TW-1	1608	MW495435
136	OT/Taiwan/KM1307b/2013	KM1307b	TW-1	1608	MW495436
137	OT/Taiwan/KM1307c/2013	KM1307c	TW-1	1608	MW495437
138	OT/Taiwan/PT1307a/2013	PT1307a	TW-1	1608	MW495438
139	OT/Taiwan/KM1308a/2013	KM1308a	TW-1	1608	MW495439
140	OT/Taiwan/NTC1307b/2013	NTC1307b	TW-1	1608	MW495440
141	OT/Taiwan/KM1310a/2013	KM1310a	TW-1	1608	MW495441
142	OT/Taiwan/PH1310a/2013	PH1310a	TW-1	1608	MW495442
143	OT/Taiwan/KHC1309a/2013	KHC1309a	TW-1	1608	MW495443
144	OT/Taiwan/KM1309c/2013	KM1309c	TW-1	1608	MW495444
145	OT/Taiwan/TT1306b/2013	TT1306b	TW-1	1608	MW495445
146	OT/Taiwan/NTC1306a/2013	NTC1306a	TW-1	1608	MW495446
147	OT/Taiwan/NTC1307a/2013	NTC1307a	TW-1	1608	MW495447

148	OT/Taiwan/TY1306a/2013	TY1306a	TW-1	1608	MW495448
149	OT/Taiwan/KM1309a/2013	KM1309a	TW-1	1608	MW495449
150	OT/Taiwan/TT1311b/2013	TT1311b	TW-1	1608	MW495450
151	OT/Taiwan/TT1309a/2013	TT1309a	TW-1	1608	MW495451
152	OT/Taiwan/TT1404b/2014	TT1404b	TW-1	1608	MW495452
153	OT/Taiwan/TT1405a/2014	TT1405a	TW-1	1608	MW495453
154	OT/Taiwan/TT1405c/2014	TT1405c	TW-1	1608	MW495454
155	OT/Taiwan/HL1406a/2014	HL1406a	TW-1	1608	MW495455
156	OT/Taiwan/CH1405c/2014	CH1405c	TW-1	1608	MW495456
157	OT/Taiwan/TT1406a/2014	TT1406a	TW-1	1608	MW495457
158	OT/Taiwan/KHC1407b/2014	KHC1407b	TW-1	1608	MW495458
159	OT/Taiwan/PH1408a/2014	PH1408a	TW-1	1608	MW495459
160	OT/Taiwan/TT1410a/2014	TT1410a	TW-1	1608	MW495460
161	OT/Taiwan/TT1411a/2014	TT1411a	TW-1	1608	MW495461
162	OT/Taiwan/TT1411b/2014	TT1411b	TW-1	1608	MW495462
163	OT/Taiwan/KM1405b/2014	KM1405b	TW-1	1608	MW495463
164	OT/Taiwan/KM1407b/2014	KM1407b	TW-1	1608	MW495464
165	OT/Taiwan/TY1406b/2014	TY1406b	TW-1	1608	MW495465
166	OT/Taiwan/NTC1407a/2014	NTC1407a	TW-1	1608	MW495466
167	OT/Taiwan/KM1409d/2014	KM1409d	TW-1	1608	MW495467
168	OT/Taiwan/KM1505a/2015	KM1505a	TW-1	1608	MW495468
169	OT/Taiwan/KM1505b/2015	KM1505b	TW-1	1608	MW495469
170	OT/Taiwan/KM1506a/2015	KM1506a	TW-1	1608	MW495470
171	OT/Taiwan/KM1506b/2015	KM1506b	TW-1	1608	MW495471
172	OT/Taiwan/KM1506c/2015	KM1506c	TW-1	1608	MW495472
173	OT/Taiwan/KM1506d/2015	KM1506d	TW-1	1608	MW495473
174	OT/Taiwan/KM1506e/2015	KM1506e	TW-1	1608	MW495474
175	OT/Taiwan/KM1506f/2015	KM1506f	TW-1	1608	MW495475
176	OT/Taiwan/KM1506g/2015	KM1506g	TW-1	1608	MW495476
177	OT/Taiwan/TY1507a/2015	TY1507a	TW-1	1608	MW495477
178	OT/Taiwan/KM1510a/2015	KM1510a	TW-1	1608	MW495478
179	OT/Taiwan/KM1510b/2015	KM1510b	TW-1	1608	MW495479
180	OT/Taiwan/KH1511a/2015	KH1511a	TW-1	1608	MW495480
181	OT/Taiwan/NTC1506a/2015	NTC1506a	TW-1	1608	MW495481
182	OT/Taiwan/HL1506b/2015	HL1506b	TW-1	1608	MW495482
183	OT/Taiwan/NTC1510b/2015	NTC1510b	TW-1	1608	MW495483
184	OT/Taiwan/KM1510c/2015	KM1510c	TW-1	1608	MW495484

185	OT/Taiwan/PH1605a/2016	PH1605a	TW-1	1608	MW495485
186	OT/Taiwan/HL1604b/2016	HL1604b	TW-1	1608	MW495486
187	OT/Taiwan/TY1605a/2016	TY1605a	TW-1	1608	MW495487
188	OT/Taiwan/TY1608a/2016	TY1608a	TW-1	1608	MW495488
189	OT/Taiwan/PH1608a/2016	PH1608a	TW-1	1608	MW495489
190	OT/Taiwan/PH1610a/2016	PH1610a	TW-1	1608	MW495490
191	OT/Taiwan/PH1609a/2016	PH1609a	TW-1	1608	MW495491
192	OT/Taiwan/TT1611a/2016	TT1611a	TW-1	1608	MW495492
193	OT/Taiwan/KM1611b/2016	KM1611b	TW-1	1608	MW495493
194	OT/Taiwan/PH1611a/2016	PH1611a	TW-1	1608	MW495494
195	OT/Taiwan/KM1610a/2016	KM1610a	TW-1	1608	MW495495
196	OT/Taiwan/KM1609a/2016	KM1609a	TW-1	1608	MW495496
197	OT/Taiwan/KM1606b/2016	KM1606b	TW-1	1608	MW495497
198	OT/Taiwan/KM1606a/2016	KM1606a	TW-1	1608	MW495498
199	OT/Taiwan /TY0610a/2016	TY0610a	TW-2	1605	GQ332743
200	OT/Taiwan/TT0906a/2009	TT0906a	TW-2	1605	MW495499
201	OT/Taiwan/TT1205a/2012	TT1205a	TW-2	1605	MW495500
202	OT/Taiwan/NTC1409a/2014	NTC1409a	TW-2	1605	MW495501
203	OT/Taiwan/TP0607a/2006	TP0607a	TW-3	1605	GQ332744
204	OT/Taiwan/TP0708a/2007	TP0708a	TW-4	1608	GQ332745
205	OT/Taiwan/NT0805a/2008	NT0805a	TW-4	1608	MW495502
206	OT/Taiwan/KM0806h/2008	KM0806h	TW-4	1608	MW495503
207	OT/Taiwan/KM1208a/2012	KM1208a	TW-4	1608	MW495504
208	OT/Taiwan/HL1407a/2014	HL1407a	TW-4	1608	MW495505
209	OT/Taiwan/HL1510a/2015	HL1510a	TW-4	1608	MW495506
210	OT/Taiwan/HL1604a/2016	HL1604a	TW-4	1608	MW495507
211	OT/Taiwan/HL1605a/2016	HL1605a	TW-4	1608	MW495508
212	OT/Taiwan/KL1607a/2016	KL1607a	TW-4	1608	MW495509
213	OT/Taiwan/KM1611a/2016	KM1611a	TW-4	1608	MW495510
214	OT/Taiwan/KM0607h/2006	KM0607h	TW-5	1632	GQ332746
215	OT/Taiwan/KM0707f/2007	KM0707f	TW-5	1632	MW495511
216	OT/Taiwan/KM0707g/2007	KM0707g	TW-5	1632	MW495512
217	OT/Taiwan/KM0710a/2007	KM0710a	TW-5	1632	MW495513
218	OT/Taiwan/KM0806d/2008	KM0806d	TW-5	1632	MW495514
219	OT/Taiwan/KM0807g/2008	KM0807g	TW-5	1632	MW495515
220	OT/Taiwan/KM1009a/2010	KM1009a	TW-5	1632	MW495516
221	OT/Taiwan/KM1407d/2014	KM1407d	TW-5	1632	MW495517

222	OT/Taiwan/KM1608c/2016	KM1608c	TW-5	1632	MW495518
223	OT/Taiwan/KHC0609c/2006	KHC0609c	TW-6	1608	GQ332747
224	OT/Taiwan/KH0705a/2007	KH0705a	TW-6	1608	MW495519
225	OT/Taiwan/YU0808a/2008	YU0808a	TW-6	1608	MW495520
226	OT/Taiwan/PT1311a/2013	PT1311a	TW-6	1608	MW495522
227	OT/Taiwan/KHC1410a/2014	KHC1410a	TW-6	1608	MW495523
228	OT/Taiwan/KHC0907a/2009	KHC0907a	TW-6	1608	MW495525
229	OT/Taiwan/KHC0606a/2006	KHC0606a	TW-7	1608	GQ332748
230	OT/Taiwan/KH0907a/2009	KH0907a	TW-7	1608	MW495521
231	OT/Taiwan/NT0807b/2008	NT0807b	TW-7	1608	MW495524
232	OT/Taiwan/TT0907b/2009	TT0907b	TW-7	1608	MW495526
233	OT/Taiwan/PT0912a/2009	PT0912a	TW-7	1608	MW495527
234	OT/Taiwan/KHC1308a/2013	KHC1308a	TW-7	1608	MW495528
235	OT/Taiwan /CH0711a/2007	CH0711a	TW-8	1692	GQ332749
236	OT/Taiwan/PH0711b/2007	PH0711b	TW-8	1692	MW495529
237	OT/Taiwan/PH0711c/2007	PH0711c	TW-8	1692	MW495530
238	OT/Taiwan/PH0811b/2008	PH0811b	TW-8	1692	MW495531
239	OT/Taiwan/PT1507a/2015	PT1507a	TW-8	1692	MW495532
240	OT/Taiwan/PH1607a/2016	PH1607a	TW-8	1692	MW495533
241	OT/Taiwan/KH1610a/2016	KH1610a	TW-8	1692	MW495534
242	OT/Taiwan/TPC0701a/2007	TPC0701a	TW-9	1599	GQ332750
243	OT/Taiwan/ML0712a/2007	ML0712a	TW-9	1599	MW495535
244	OT/Taiwan/TCC0712a/2007	TCC0712a	TW-9	1599	MW495536
245	OT/Taiwan/TC0712a/2007	TC0712a	TW-9	1599	MW495537
246	OT/Taiwan/NT0712b/2007	NT0712b	TW-9	1599	MW495538
247	OT/Taiwan/NT0712a/2007	NT0712a	TW-9	1599	MW495539
248	OT/Taiwan/NT0711e/2007	NT0711e	TW-9	1599	MW495540
249	OT/Taiwan/NT0711b/2007	NT0711b	TW-9	1599	MW495541
250	OT/Taiwan/HC0711a/2007	HC0711a	TW-9	1599	MW495542
251	OT/Taiwan/KHC0711a/2007	KHC0711a	TW-9	1599	MW495543
252	OT/Taiwan/HC0711b/2007	HC0711b	TW-9	1599	MW495544
253	OT/Taiwan/TY0711a/2007	TY0711a	TW-9	1599	MW495545
254	OT/Taiwan/TY0712a/2007	TY0712a	TW-9	1599	MW495546
255	OT/Taiwan/ML0712b/2007	ML0712b	TW-9	1599	MW495547
256	OT/Taiwan/TY0712b/2007	TY0712b	TW-9	1599	MW495548
257	OT/Taiwan/HC0712a/2007	HC0712a	TW-9	1599	MW495549
258	OT/Taiwan/TP0712a/2007	TP0712a	TW-9	1599	MW495550

259	OT/Taiwan/KL0802a/2008	KL0802a	TW-9	1599	MW495551
260	OT/Taiwan/ML0801a/2008	ML0801a	TW-9	1599	MW495552
261	OT/Taiwan/HC0801a/2008	HC0801a	TW-9	1599	MW495553
262	OT/Taiwan/TP0801a/2008	TP0801a	TW-9	1599	MW495554
263	OT/Taiwan/TPC0801a/2008	TPC0801a	TW-9	1599	MW495555
264	OT/Taiwan/NT0812a/2008	NT0812a	TW-9	1599	MW495556
265	OT/Taiwan/NT0812b/2008	NT0812b	TW-9	1599	MW495557
266	OT/Taiwan/TCC0812a/2008	TCC0812a	TW-9	1599	MW495558
267	OT/Taiwan/NT0801a/2008	NT0801a	TW-9	1599	MW495559
268	OT/Taiwan/NT0801b/2008	NT0801b	TW-9	1599	MW495560
269	OT/Taiwan/TP0811a/2008	TP0811a	TW-9	1599	MW495561
270	OT/Taiwan/TY0812a/2008	TY0812a	TW-9	1599	MW495562
271	OT/Taiwan/TC0812a/2008	TC0812a	TW-9	1599	MW495563
272	OT/Taiwan/TY0812b/2008	TY0812b	TW-9	1599	MW495564
273	OT/Taiwan/ML0812a/2008	ML0812a	TW-9	1599	MW495565
274	OT/Taiwan/TPC0812a/2008	TPC0812a	TW-9	1599	MW495566
275	OT/Taiwan/TT0902a/2009	TT0902a	TW-9	1599	MW495567
276	OT/Taiwan/HL0901a/2009	HL0901a	TW-9	1599	MW495568
277	OT/Taiwan/HL0902a/2009	HL0902a	TW-9	1599	MW495569
278	OT/Taiwan/TC0912a/2009	TC0912a	TW-9	1599	MW495570
279	OT/Taiwan/KL0902a/2009	KL0902a	TW-9	1599	MW495571
280	OT/Taiwan/TPC0902a/2009	TPC0902a	TW-9	1599	MW495572
281	OT/Taiwan/TP0912a/2009	TP0912a	TW-9	1599	MW495573
282	OT/Taiwan/TP1003a/2010	TP1003a	TW-9	1599	MW495574
283	OT/Taiwan/TP1101a/2011	TP1101a	TW-9	1599	MW495575
284	OT/Taiwan/TCC1101a/2011	TCC1101a	TW-9	1599	MW495576
285	OT/Taiwan/HL1201a/2012	HL1201a	TW-9	1599	MW495577
286	OT/Taiwan/ML1412a/2014	ML1412a	TW-9	1599	MW495578
287	OT/Taiwan/TT1412a/2014	TT1412a	TW-9	1599	MW495579
288	OT/Taiwan/TT1501a/2015	TT1501a	TW-9	1599	MW495580
289	OT/Taiwan/TT1502a/2015	TT1502a	TW-9	1599	MW495581
290	OT/Taiwan/KHC0704a/2007	KHC0704a	TW-10	1566	GQ332751
291	OT/Taiwan/KL0807a/2008	KL0807a	TW-10	1566	MW495582
292	OT/Taiwan/TT0808b/2008	TT0808b	TW-10	1566	MW495583
293	OT/Taiwan/TT0807b/2008	TT0807b	TW-10	1566	MW495584
294	OT/Taiwan/HL0906b/2009	HL0906b	TW-10	1566	MW495585
295	OT/Taiwan/TT1005c/2010	TT1005c	TW-10	1566	MW495586

296	OT/Taiwan/TT1012a/2010	TT1012a	TW-10	1566	MW495587
297	OT/Taiwan/TT1106a/2011	TT1106a	TW-10	1566	MW495588
298	OT/Taiwan/HL1205a/2012	HL1205a	TW-10	1566	MW495589
299	OT/Taiwan/HL1312a/2013	HL1312a	TW-10	1566	MW495590
300	OT/Taiwan/TT1404a/2014	TT1404a	TW-10	1566	MW495591
301	OT/Taiwan/TY1406a/2014	TY1406a	TW-10	1566	MW495592
302	OT/Taiwan/KHC1405a/2014	KHC1405a	TW-10	1566	MW495593
303	OT/Taiwan/KH1508a/2015	KH1508a	TW-10	1566	MW495594
304	OT/Taiwan/NTC1510a/2015	NTC1510a	TW-10	1566	MW495595
305	OT/Taiwan/TP1608a/2016	TP1608a	TW-10	1566	MW495596
306	OT/Taiwan/NTC1611a/2016	NTC1611a	TW-10	1566	MW495597
307	OT/Taiwan/HL1612a/2016	HL1612a	TW-10	1566	MW495598
308	OT/Taiwan/NT0707a/2007	NT0707a	TW-11	1584	GQ332752
309	OT/Taiwan/ TT0705a /2007	TT0705a	TW-12	1593	GQ332753
310	OT/Taiwan/TT1006a/2010	TT1006a	TW-12	1593	MW495599
311	OT/Taiwan/NT0711a/2007	NT0711a	TW-13	1557	GQ332754
312	OT/Taiwan/NT0806a/2008	NT0806a	TW-13	1557	MW495600
313	OT/Taiwan/NT0807a/2008	NT0807a	TW-13	1557	MW495601
314	OT/Taiwan/TT0711a/2007	TT0711a	TW-14	1551	GQ332755
315	OT/Taiwan/PT0712b/2007	PT0712b	TW-15	1569	GQ332756
316	OT/Taiwan/KHC0707a/2007	KHC0707a	TW-16	1572	GQ332757
317	OT/Taiwan/TT0802a/2008	TT0802a	TW-16	1572	MW495602
318	OT/Taiwan/LC0807d/2008	LC0807d	TW-16	1572	MW495603
319	OT/Taiwan/TY0807a/2008	TY0807a	TW-16	1572	MW495604
320	OT/Taiwan/LC0808a/2008	LC0808a	TW-16	1572	MW495605
321	OT/Taiwan/LC0808c/2008	LC0808c	TW-16	1572	MW495606
322	OT/Taiwan/LC0908a/2009	LC0908a	TW-16	1572	MW495607
323	OT/Taiwan/TT1005d/2010	TT1005d	TW-16	1572	MW495608
324	OT/Taiwan/TC1008a/2010	TC1008a	TW-16	1572	MW495609
325	OT/Taiwan/TT1310a/2013	TT1310a	TW-16	1572	MW495610
326	OT/Taiwan/PH1509a/2015	PH1509a	TW-16	1572	MW495611
327	OT/Taiwan/TPC0707a/2007	TPC0707a	TW-17	1596	GQ332758
328	OT/Taiwan/KM1407c/2014	KM1407c	TW-17	1596	MW495612
329	OT/Taiwan/KHC0706a/2007	KHC0706a	TW-18	1596	GQ332759
330	OT/Taiwan/ML0711a/2007	ML0711a	TW-18	1596	MW495613
331	OT/Taiwan/KHC1411a/2014	KHC1411a	TW-18	1596	MW495614
332	OT/Taiwan/KH1607a/2016	KH1607a	TW-18	1596	MW495615

333	OT/Taiwan/PT0909a/2009	PT0909a	TW-18	1572	MW495616
334	OT/Taiwan/KM0606a/2006	KM0606a	TW-19	1572	GQ332760
335	OT/Taiwan/NT0607b/2006	NT0607b	TW-19	1572	MW495617
336	OT/Taiwan/KHC0608c/2006	KHC0608c	TW-19	1572	MW495618
337	OT/Taiwan/KHC0609a/2006	KHC0609a	TW-19	1572	MW495619
338	OT/Taiwan/LC0608a/2006	LC0608a	TW-19	1572	MW495620
339	OT/Taiwan/NT0607a/2006	NT0607a	TW-19	1572	MW495621
340	OT/Taiwan/KM0706h/2007	KM0706h	TW-19	1572	MW495622
341	OT/Taiwan/KM0706g/2007	KM0706g	TW-19	1572	MW495623
342	OT/Taiwan/TC0705a/2007	TC0705a	TW-19	1572	MW495624
343	OT/Taiwan/NT0705a/2007	NT0705a	TW-19	1572	MW495625
344	OT/Taiwan/PT0708a/2007	PT0708a	TW-19	1572	MW495626
345	OT/Taiwan/NT0711c/2007	NT0711c	TW-19	1572	MW495627
346	OT/Taiwan/NT0711d/2007	NT0711d	TW-19	1572	MW495628
347	OT/Taiwan/NT0711f/2007	NT0711f	TW-19	1572	MW495629
348	OT/Taiwan/KM0805a/2008	KM0805a	TW-19	1572	MW495630
349	OT/Taiwan/KH0807a/2008	KH0807a	TW-19	1572	MW495631
350	OT/Taiwan/NT0808a/2008	NT0808a	TW-19	1572	MW495632
351	OT/Taiwan/TN0809a/2008	TN0809a	TW-19	1572	MW495633
352	OT/Taiwan/KM0809e/2008	KM0809e	TW-19	1572	MW495634
353	OT/Taiwan/HL0903a/2009	HL0903a	TW-19	1572	MW495635
354	OT/Taiwan/KM0907h/2009	KM0907h	TW-19	1572	MW495636
355	OT/Taiwan/HL0907a/2009	HL0907a	TW-19	1572	MW495637
356	OT/Taiwan/NT0907a/2009	NT0907a	TW-19	1572	MW495638
357	OT/Taiwan/TT0907c/2009	TT0907c	TW-19	1572	MW495639
358	OT/Taiwan/HL0909b/2009	HL0909b	TW-19	1572	MW495640
359	OT/Taiwan/KHC0909a/2009	KHC0909a	TW-19	1572	MW495641
360	OT/Taiwan/KM0909b/2009	KM0909b	TW-19	1572	MW495642
361	OT/Taiwan/HL0911b/2009	HL0911b	TW-19	1572	MW495643
362	OT/Taiwan/CH0911a/2009	CH0911a	TW-19	1572	MW495644
363	OT/Taiwan/TP0911a/2009	TP0911a	TW-19	1572	MW495645
364	OT/Taiwan/HL1004a/2010	HL1004a	TW-19	1572	MW495646
365	OT/Taiwan/TC1004a/2010	TC1004a	TW-19	1572	MW495647
366	OT/Taiwan/TCC1004a/2010	TCC1004a	TW-19	1572	MW495648
367	OT/Taiwan/HL1006a/2010	HL1006a	TW-19	1572	MW495649
368	OT/Taiwan/CH1006a/2010	CH1006a	TW-19	1572	MW495650
369	OT/Taiwan/HL1003a/2010	HL1003a	TW-19	1572	MW495651

370	OT/Taiwan/TT1004b/2010	TT1004b	TW-19	1572	MW495652
371	OT/Taiwan/NT1107a/2011	NT1107a	TW-19	1572	MW495653
372	OT/Taiwan/ML1107a/2011	ML1107a	TW-19	1572	MW495654
373	OT/Taiwan/TT1110a/2011	TT1110a	TW-19	1572	MW495655
374	OT/Taiwan/TT1303a/2013	TT1303a	TW-19	1572	MW495656
375	OT/Taiwan/HL1306a/2013	HL1306a	TW-19	1572	MW495657
376	OT/Taiwan/HL1307a/2013	HL1307a	TW-19	1572	MW495658
377	OT/Taiwan/TT1311a/2013	TT1311a	TW-19	1572	MW495659
378	OT/Taiwan/HL1311a/2013	HL1311a	TW-19	1572	MW495660
379	OT/Taiwan/HL1312b/2013	HL1312b	TW-19	1572	MW495661
380	OT/Taiwan/CH1405a/2014	CH1405a	TW-19	1572	MW495662
381	OT/Taiwan/KM1409b/2014	KM1409b	TW-19	1572	MW495663
382	OT/Taiwan/KM1409e/2014	KM1409e	TW-19	1572	MW495664
383	OT/Taiwan/TPC1411a/2014	TPC1411a	TW-19	1572	MW495665
384	OT/Taiwan/HL1504a/2015	HL1504a	TW-19	1572	MW495666
385	OT/Taiwan/LC1507a/2015	LC1507a	TW-19	1572	MW495667
386	OT/Taiwan/NT1511a/2015	NT1511a	TW-19	1572	MW495668
387	OT/Taiwan/HL1609a/2016	HL1609a	TW-19	1572	MW495669
388	OT/Taiwan/HC0605a/2006	HC0605a	TW-20	1572	GQ332761
389	OT/Taiwan/KHC0705a/2007	KHC0705a	TW-20	1572	MW495670
390	OT/Taiwan/HL0908a/2009	HL0908a	TW-20	1572	MW495671
391	OT/Taiwan/KL1309a/2013	KL1309a	TW-20	1572	MW495672
392	OT/Taiwan/HL1507a/2015	HL1507a	TW-20	1572	MW495673
393	OT/Taiwan/KM0607b/2006	KM0607b	TW-21	1590	GQ332762
394	OT/Taiwan/KM0609a/2006	KM0609a	TW-21	1590	MW495674
395	OT/Taiwan/KM0806e/2008	KM0806e	TW-21	1590	MW495675
396	OT/Taiwan/KM0907a/2009	KM0907a	TW-21	1590	MW495676
397	OT/Taiwan/KM1409a/2014	KM1409a	TW-21	1590	MW495677
398	OT/Taiwan/NTC1510d/2015	NTC1510d	TW-21	1590	MW495678
399	OT/Taiwan/KHC0606b/2006	KHC0606b	TW-22	1575	GQ332763
400	OT/Taiwan/KH0609a/2006	KH0609a	TW-22	1575	MW495679
401	OT/Taiwan/KHC0609b/2006	KHC0609b	TW-22	1575	MW495680
402	OT/Taiwan/KHC0608a/2006	KHC0608a	TW-22	1575	MW495681
403	OT/Taiwan/KH0610a/2006	KH0610a	TW-22	1575	MW495682
404	OT/Taiwan/KM0609b/2006	KM0609b	TW-22	1575	MW495683
405	OT/Taiwan/KH0609b/2006	KH0609b	TW-22	1575	MW495684
406	OT/Taiwan/KHC0608b/2006	KHC0608b	TW-22	1575	MW495685

407	OT/Taiwan/KHC0707b/2007	KHC0707b	TW-22	1575	MW495686
408	OT/Taiwan/KM0706f/2007	KM0706f	TW-22	1575	MW495687
409	OT/Taiwan/KM0706a/2007	KM0706a	TW-22	1575	MW495688
410	OT/Taiwan/KM0705b/2007	KM0705b	TW-22	1575	MW495689
411	OT/Taiwan/KM0707e/2007	KM0707e	TW-22	1575	MW495690
412	OT/Taiwan/LC0707a/2007	LC0707a	TW-22	1575	MW495691
413	OT/Taiwan/KM0707j/2007	KM0707j	TW-22	1575	MW495692
414	OT/Taiwan/KHC0710a/2007	KHC0710a	TW-22	1575	MW495693
415	OT/Taiwan/KM0710b/2007	KM0710b	TW-22	1575	MW495694
416	OT/Taiwan/KM0709a/2007	KM0709a	TW-22	1575	MW495695
417	OT/Taiwan/KHC0709a/2007	KHC0709a	TW-22	1575	MW495696
418	OT/Taiwan/KHC0708a/2007	KHC0708a	TW-22	1575	MW495697
419	OT/Taiwan/KHC0807b/2008	KHC0807b	TW-22	1575	MW495698
420	OT/Taiwan/KHC0807c/2008	KHC0807c	TW-22	1575	MW495699
421	OT/Taiwan/KM0807i/2008	KM0807i	TW-22	1575	MW495700
422	OT/Taiwan/KHC0807a/2008	KHC0807a	TW-22	1575	MW495701
423	OT/Taiwan/CH0808a/2008	CH0808a	TW-22	1575	MW495702
424	OT/Taiwan/KM0809a/2008	KM0809a	TW-22	1575	MW495703
425	OT/Taiwan/KM0810a/2008	KM0810a	TW-22	1575	MW495704
426	OT/Taiwan/TNC0811a/2008	TNC0811a	TW-22	1575	MW495705
427	OT/Taiwan/KM0808a/2008	KM0808a	TW-22	1575	MW495706
428	OT/Taiwan/KM0808b/2008	KM0808b	TW-22	1575	MW495707
429	OT/Taiwan/KHC0807d/2008	KHC0807d	TW-22	1575	MW495708
430	OT/Taiwan/KHC0809a/2008	KHC0809a	TW-22	1575	MW495709
431	OT/Taiwan/KHC0808a/2008	KHC0808a	TW-22	1575	MW495710
432	OT/Taiwan/KM0907f/2009	KM0907f	TW-22	1575	MW495711
433	OT/Taiwan/KM0907g/2009	KM0907g	TW-22	1575	MW495712
434	OT/Taiwan/KH0911a/2009	KH0911a	TW-22	1575	MW495713
435	OT/Taiwan/KM1006c/2010	KM1006c	TW-22	1575	MW495714
436	OT/Taiwan/KM1007a/2010	KM1007a	TW-22	1575	MW495715
437	OT/Taiwan/KM1007b/2010	KM1007b	TW-22	1575	MW495716
438	OT/Taiwan/TP1007a/2010	TP1007a	TW-22	1575	MW495717
439	OT/Taiwan/KHC1009b/2010	KHC1009b	TW-22	1575	MW495718
440	OT/Taiwan/TT1004a/2010	TT1004a	TW-22	1575	MW495719
441	OT/Taiwan/TN1005a/2010	TN1005a	TW-22	1575	MW495720
442	OT/Taiwan/KHC1009a/2010	KHC1009a	TW-22	1575	MW495721
443	OT/Taiwan/TT1009a/2010	TT1009a	TW-22	1575	MW495722

444	OT/Taiwan/HL1011b/2010	HL1011b	TW-22	1575	MW495723
445	OT/Taiwan/KH0907b/2009	KH0907b	TW-22	1575	MW495724
446	OT/Taiwan/TT1005b/2010	TT1005b	TW-22	1575	MW495725
447	OT/Taiwan/TY1110a/2011	TY1110a	TW-22	1575	MW495726
448	OT/Taiwan/KM1106f/2011	KM1106f	TW-22	1575	MW495727
449	OT/Taiwan/KM1206b/2012	KM1206b	TW-22	1575	MW495728
450	OT/Taiwan/KM1206c/2012	KM1206c	TW-22	1575	MW495729
451	OT/Taiwan/KM1206e/2012	KM1206e	TW-22	1575	MW495730
452	OT/Taiwan/KM1207a/2012	KM1207a	TW-22	1575	MW495731
453	OT/Taiwan/NTC1206a/2012	NTC1206a	TW-22	1575	MW495732
454	OT/Taiwan/HL1209a/2012	HL1209a	TW-22	1575	MW495733
455	OT/Taiwan/TT1212a/2012	TT1212a	TW-22	1575	MW495734
456	OT/Taiwan/KH1305a/2013	KH1305a	TW-22	1575	MW495735
457	OT/Taiwan/TT1308a/2013	TT1308a	TW-22	1575	MW495736
458	OT/Taiwan/KHC1310a/2013	KHC1310a	TW-22	1575	MW495737
459	OT/Taiwan/KHC1309b/2013	KHC1309b	TW-22	1575	MW495738
460	OT/Taiwan/KHC1307a/2013	KHC1307a	TW-22	1575	MW495739
461	OT/Taiwan/KM1405a/2014	KM1405a	TW-22	1575	MW495740
462	OT/Taiwan/KHC1406a/2014	KHC1406a	TW-22	1575	MW495741
463	OT/Taiwan/KM1406a/2014	KM1406a	TW-22	1575	MW495742
464	OT/Taiwan/TY1406c/2014	TY1406c	TW-22	1575	MW495743
465	OT/Taiwan/KM1407a/2014	KM1407a	TW-22	1575	MW495744
466	OT/Taiwan/PT1407a/2014	PT1407a	TW-22	1575	MW495745
467	OT/Taiwan/KHC1407a/2014	KHC1407a	TW-22	1575	MW495746
468	OT/Taiwan/KHC1408a/2014	KHC1408a	TW-22	1575	MW495747
469	OT/Taiwan/KM1409c/2014	KM1409c	TW-22	1575	MW495748
470	OT/Taiwan/TT1405d/2014	TT1405d	TW-22	1575	MW495749
471	OT/Taiwan/KHC1406b/2014	KHC1406b	TW-22	1575	MW495750
472	OT/Taiwan/TT1410b/2014	TT1410b	TW-22	1575	MW495751
473	OT/Taiwan/KHC1410b/2014	KHC1410b	TW-22	1575	MW495752
474	OT/Taiwan/HC1509a/2015	HC1509a	TW-22	1575	MW495753
475	OT/Taiwan/PT1509a/2015	PT1509a	TW-22	1575	MW495754
476	OT/Taiwan/KH1510a/2015	KH1510a	TW-22	1575	MW495755
477	OT/Taiwan/CY1510a/2015	CY1510a	TW-22	1575	MW495756
478	OT/Taiwan/TT1503a/2015	TT1503a	TW-22	1575	MW495757
479	OT/Taiwan/NTC1510c/2015	NTC1510c	TW-22	1575	MW495758
480	OT/Taiwan/KH1509a/2015	KH1509a	TW-22	1575	MW495759

481	OT/Taiwan/PT1604a/2016	PT1604a	TW-22	1575	MW495760
482	OT/Taiwan/KH1605b/2016	KH1605b	TW-22	1575	MW495761
483	OT/Taiwan/KH1608b/2016	KH1608b	TW-22	1575	MW495762
484	OT/Taiwan/KM1608b/2016	KM1608b	TW-22	1575	MW495763
485	OT/Taiwan/TCC1610a/2016	TCC1610a	TW-22	1575	MW495764
486	OT/Taiwan/KM1610b/2016	KM1610b	TW-22	1575	MW495765
487	OT/Taiwan/KH1611a/2016	KH1611a	TW-22	1575	MW495766
488	OT/Taiwan/KM1611c/2016	KM1611c	TW-22	1575	MW495767
489	OT/Taiwan/TT1604a/2016	TT1604a	TW-22	1575	MW495768
490	OT/Taiwan/KH1608a/2016	KH1608a	TW-22	1575	MW495769
491	OT/Taiwan/KH1605c/2016	KH1605c	TW-22	1575	MW495770
492	OT/Taiwan/KH1606a/2016	KH1606a	TW-22	1575	MW495771
493	OT/Taiwan/TN1606a/2016	TN1606a	TW-22	1575	MW495772
494	OT/Taiwan/HL1609b/2016	HL1609b	TW-22	1575	MW495773
495	OT/Taiwan/KH1612a/2016	KH1612a	TW-22	1575	MW495774
496	OT/Taiwan/KM0806c/2008	KM0806c	TW-23	1611	MW460713
497	OT/Taiwan/KM0807k/2008	KM0807k	TW-23	1611	MW495775
498	OT/Taiwan/KM0906a/2009	KM0906a	TW-23	1611	MW495776
499	OT/Taiwan/KM1509a/2015	KM1509a	TW-23	1611	MW495777
500	OT/Taiwan/TN0807a/2008	TN0807a	TW-24	1602	MW464199
501	OT/Taiwan/TT0807a/2008	TT0807a	TW-24	1602	MW495778
502	OT/Taiwan/TNC0809a/2008	TNC0809a	TW-24	1602	MW495779
503	OT/Taiwan/TY0904a/2009	TY0904a	TW-24	1602	MW495780
504	OT/Taiwan/TT1010b/2010	TT1010b	TW-24	1602	MW495781
505	OT/Taiwan/TT1107b/2011	TT1107b	TW-24	1602	MW495782
506	OT/Taiwan/KHC1205a/2012	KHC1205a	TW-24	1602	MW495783
507	OT/Taiwan/TT1405b/2014	TT1405b	TW-24	1602	MW495784
508	OT/Taiwan/NTC1605a/2016	NTC1605a	TW-24	1602	MW495785
509	OT/Taiwan/TCC1605a/2016	TCC1605a	TW-24	1602	MW495786
510	OT/Taiwan/KH1605a/2016	KH1605a	TW-24	1602	MW495787
511	OT/Taiwan/TY1606a/2016	TY1606a	TW-24	1602	MW495788
512	OT/Taiwan/TT0908a/2009	TT0908a	TW-25	1605	MW464200
513	OT/Taiwan/NT1011a/2010	NT1011a	TW-25	1605	MW495789
514	OT/Taiwan/NT1310a/2013	NT1310a	TW-25	1605	MW495790
515	OT/Taiwan/TT0910a/2009	TT0910a	TW-26	1605	MW464201
516	OT/Taiwan/HL0911c/2009	HL0911c	TW-26	1605	MW495791
517	OT/Taiwan/HL0911a/2009	HL0911a	TW-26	1605	MW495792

518	OT/Taiwan/HL1009a/2010	HL1009a	TW-26	1605	MW495793
519	OT/Taiwan/HL1010a/2010	HL1010a	TW-26	1605	MW495794
520	OT/Taiwan/TPC1007b/2010	TPC1007b	TW-26	1605	MW495795
521	OT/Taiwan/KM0807h/2008	KM0807h	TW-27	1605	MW464202
522	OT/Taiwan/TT1310b/2013	TT1310b	TW-27	1605	MW495796
523	OT/Taiwan/LC1310a/2013	LC1310a	TW-27	1605	MW495797
524	OT/Taiwan/KM1309b/2013	KM1309b	TW-27	1605	MW495798
525	OT/Taiwan/KHC1409a/2014	KHC1409a	TW-27	1605	MW495799
526	OT/Taiwan/TPC0911a/2009	TPC0911a	TW-28	1587	MW464203
527	OT/Taiwan/TT1008a/2010	TT1008a	TW-28	1587	MW495800
528	OT/Taiwan/TT1211a/2012	TT1211a	TW-28	1587	MW495801
529	OT/Taiwan/CH0807a/2008	CH0807a	TW-29	1575	MW464204
530	OT/Taiwan/HC0807a/2008	HC0807a	TW-29	1575	MW495802
531	OT/Taiwan/TY1410a/2014	TY1410a	TW-29	1575	MW495803
532	OT/Taiwan/TT1506a/2015	TT1506a	TW-29	1575	MW495804
533	OT/Taiwan/HL1004b/2010	HL1004b	TW-30	1572	MW464205
534	OT/Taiwan/HL1011a/2010	HL1011a	TW-30	1572	MW495808
535	OT/Taiwan/HL1506a/2015	HL1506a	TW-30	1572	MW495809
536	OT/Taiwan/NT1211a/2012	NT1211a	TW-31	1602	MW495810
537	OT/Taiwan/YL1409a/2014	YL1409a	TW-31	1602	MW495811
538	OT/Taiwan/ML1307a/2013	ML1307a	TW-32	1599	MW495812
539	OT/Taiwan/CY1508a/2015	CY1508a	TW-32	1608	MW495813
540	OT/Taiwan/CH1405b/2014	CH1405b	TW-33	1602	MW495814
541	OT/Taiwan/TCC1505a/2015	TCC1505a	TW-34	1599	MW495815
542	OT/Taiwan/NT1610a/2016	NT1610a	TW-35	1608	MW495816
543	OT/Taiwan/NT1008a/2010	NT1008a	TW-36	1581	MW495805
544	OT/Taiwan/CY1409a/2014	CY1409a	TW-36	1575	MW495806
545	OT/Taiwan/KM1608a/2016	KM1608a	TW-36	1575	MW495807
546	OT/India/0809aTw/2008			1599	MW495817
