# Supplementary material for: Molecular epidemiology of scrub typhus in Taiwan during 2006–2016
Source: PLoS Negl Trop Dis. 2022 Apr 29;16(4):e0010369. doi: 10.1371/journal.pntd.0010369 (PMC9094550; doi:10.1371/journal.pntd.0010369)
Supplement: Attachment — Submitted filename: Response Letter .pdf [file pntd.0010369.s002.pdf]

Dear Editor: Feb. 16, 2022

We are submitting the revised manuscript (original number **PNTD-D-21-01603**) "Molecular epidemiology of scrub typhus in Taiwan during 2006-2016" by Hsiang-Fei Chen, Shih-Huan Peng, Kun-Hsien Tsai, Cheng-Fen Yang, Mei-Chun Chang, Yeou-Lin Hsueh, Chien-Ling Su, Ruo-Yu Wang, Pei-Yun Shu, Su-Lin Yang for consideration of publication in the PLOS Neglected Tropical Diseases.

We appreciate the thoughtful and constructive comments and suggestions provided by the editor and reviewers and have carefully revised the manuscript accordingly. Please refer to the following pages for our responses to the comments from the reviewers. Your careful consideration is greatly appreciated.

We hereby declare that the manuscript has not been submitted or accepted for publication elsewhere. All authors have seen and agree with the contents of the manuscript and do not have commercial or other associations that might pose a conflict of interest.

Sincerely,

Su-Lin Yang and coauthors

Center for Diagnostics and Vaccine Development,
Centers for Disease Control,
Ministry of Health and Welfare, Taiwan, R.O.C.

Method Comment

Reviewer #1:

The methods are adequate to address the issue of genetic diversity of Orientia tsutsugamushi and isolates obtain from persons with scrub typhus in Taiwan.

The 56 kDa surface protein contains four hydrophilic hypervariable regions that are the apparent mechanism of antigenic diversity of Orientia tsutsugamushi. A better understanding of the evolution and phylogeny of Orentia tsutsugamushi would be better analyzed with conserved housekeeping genes.

Response: We agree with the Reviewer's comment that investigation of the DNA sequence variations in housekeeping genes of *O. tsutsugamushi* is a promising approach to the analysis of its evolution and phylogeny of *O. tsutsugamushi*. We have described the Reviewer's suggestion in the Discussion.

The revised description in the discussion is as follows*:*

"The immunodominant 56 kDa surface protein (TSA56) is a major surface protein that contains hypervariable regions and exhibits remarkable sequence variation in different strains. Analysis of the genetic relationship of *Orientia* strains using DNA sequences of the TSA56 gene may have exaggerated the differences in the evolution of these strains. However, in the past few years, TSA56 gene of *O. tsutsugamushi* has been the target for phylogenetic analysis because of its sequence variation. It is known that the sequence diversity within housekeeping genes is very restricted, leading to the analysis of conserved housekeeping genes by multilocus sequencing (MLS), which might be required for surveillance of genetic phylogeny. Using housekeeping genes as an alternative approach to study the evolution and phylogeny of *O. tsutsugamushi* is required for future comparisons of the present results."

Reviewer #2: The methods in this study are suitable.

Result Comments

Reviewer #1:

1. The establishment of 545 isolates Orientia tsutsugamushi from confirmed cases of scrub typhus and sequencing of the 56 kilodalton surface protein gene in all of them is a major achievement.
The criterion of 98% or greater sequence similarity as determining a distinct sequence type seems arbitrary. What is the rationale for this percent.

Response: Our previous study set the cutoff value at 99.9% of *tsa56* sequence similarity for the phylogenetic analysis of 116 isolates collected during 2006-2007 in Taiwan (Phylogenetic Analysis of 56-kDa Type-Specific Antigen Gene of Orientia tsutsugamushi Isolates in Taiwan. Am. J. Med. Hyg,2010 83(3) P658-663).

In the present study, we set the criterion at 98% for 545 isolates. The criterion declined from 99.9% to 98% based on the number of isolates, thereby benefiting the analysis of phylogenetic relationships

2. Lines 214-215: Liver dysfunction is listed as a symptom. Measurement of liver function is determined by laboratory tests and is not a patient complaint. It is unlikely that these patients had hepatic dysfunction; it is much more likely that they manifested hepatic injury by elevated transaminase enzymes. Was a measurement of hepatic function such as serum ammonium concentration or intrahepatic cholestasis identified?

Response: The description of liver abnormality in Lines 214-215 came from the clinicians who reviewed the clinical data of biochemical tests, including blood levels of alanine transaminase and aspartate transaminase, as well as the clinical signs of liver dysfunction based on physical examination.

Reviewer #2: The results in this study are good and suitable.

Conclusions Comments

Reviewer #1:

1. On lines 290 and 296 the issue of antigenic variation is raised. The data in the paper are gene sequences, i.e. genotypes. It is very likely that the most important antigenic variation of Orientia tsutsugamushi is determined by the four hypervariable regions of the 56 kDa surface protein. Knowledge of the genotype does not provide any information about the serotypes, which are very likely highly variable within the Karp group genotype. The reviewer is unaware of any correlation between serotypes and genotypes of Orientia tsutsugamushi. The situation is not clearly stated in this manuscript.

Response: We thank the reviewer for pointing this out. Serotyping has been used to classify a new isolate of *O. tsutsugamushi* based on reactivity with strain- or type-specific monoclonal antibodies or with hyperimmune sera recognizing a specific motif on TSA56 from well-characterized strains. However, serotyping often exhibits moderate cross-reactivity between an unidentified isolate and prototype strains (or well-established strains). In contrast, genotyping of TSA56 is a promising approach to determine the molecular epidemiology of *O. tsutsugamushi.* For a better understanding of the correlation between serotypes and genotypes of O. tsutsugamushi, it would be valuable to extensively investigate the serotypes of O. tsutsugamushi among our isolated strains and evaluate their performance in serological diagnosis in the future.

The revised description in the Discussion is as follows:

"Serotyping has been used to classify a new isolate of *O. tsutsugamushi* based on reactivity with strain- or type-specific monoclonal antibodies or hyperimmune sera recognizing a specific motif on TSA56 from well-characterized strains [34]. However, serotyping often exhibits moderate cross-reactivity between the unidentified isolate and prototype strains. In contrast, genotyping of TSA56 is a promising approach to determine the molecular epidemiology of *O. tsutsugamushi*. Therefore, we sequenced the complete TSA56 gene of 545 isolates to analyze the genetic diversity of *O. tsutsugamushi* during the 2006-2016 surveillance in Taiwan. Nevertheless, for a better understanding of the correlation between serotypes and genotypes of *O. tsutsugamushi*, it would be valuable to extensively investigate the serotypes of *O. tsutsugamushi* among our isolates in the future."

2. Lines 249-250: There is no evidence that animal hosts of the chiggers determine variation in Orientia tsutsugamushi. Rodents are hosts of the chiggers but are not hosts of Orientia tsutsugamushi. Although rodents are infected with Orientia tsutsugamushi and chiggers can become infected while feeding on an infected rodent, these chiggers do not transmit the bacteria transovarially. Chiggers are the only true host of Orientia tsutsugamushi. Animals are dead-end hosts.

Response: Thank you. We agree that chiggers are the only host of *Orientia tsutsugamushi*. The word "rodent" has been removed, and the sentence has been modified.

The revised description is as follows*:*

"This may be ascribed to the variation of the vectors, at least in part, being controlled by geographical and seasonal factors. Wang and colleagues reported various chiggers linked to geographical and seasonal variation that act as potential vectors, leading to the formation of a dominant *O. tsutsugamushi* transmission chain in Taiwan [22]. In addition, invasive plants change the survival of certain vectors and affect the transmission of *O. tsutsugamushi* [23]."

3. Lines 272-275: Are the severe manifestations in patients infected with TW-1 and TW-2 statistically significantly different from the manifestations in other strains? Are the mild syndromes observed in patients with TW-3, TW-4, TW-5, and TW-17 statistically different compared with patients infected with other strains?

Response: It was the tendency that TW-1 and TW-22 generally had more severe complications, and TW-3, TW-14, TW-15, and TW-17 usually had mild symptoms, but there were no statistically significant differences (P>0.05, Fisher's exact test). Therefore, we decided to remove Lines 272-275.

Reviewer #2: The conclusions are reasonable and suitable.

Editorial and Data Presentation Modifications

Reviewer #1: I believe that the authors should address the issues that I have raised under conclusions, results, and methods.

Response: Thank you. We have responded to comments on methods, results and conclusions in the revised manuscript

Reviewer #2: Major Revision.
Please see "Summary and General Comments".

Response: Thank you. We have responded to commends on summary and general comments section.

Summary and General Comments
Reviewer #1: This manuscript presents outstanding data and merits publication.
Response: We thank the Reviewer for the comments.

Reviewer #2: Authors studied the tsa56 genotypes of 545 O. tsutsugamushi clinical isolates throughout Taiwan, including offshore islands, from 2006 to 2016 to elucidate the molecular epidemiology of scrub typhus in Taiwan and data indicate the widespread presence of tsa56 genotypes closely related to Thailand and Korean strains and the presence of the unique endemic strains TW-12, TW-22, TW-29, and TW-36 in Taiwan.
1. Several reports reported mixed (or co) infection of different genotypes in a patient in SE and East Asia
1. Emerg Infect Dis. 2018 Aug;24(8):1520-1523. doi: 10.3201/eid2408.171622. Dual Genotype Orientia tsutsugamushi Infection in Patient with Rash and Eschar, Vietnam, 2016.
2. Am. J. Trop. Med. Hyg., 99(2), 2018, pp. 287–290. doi:10.4269/ajtmh.18-0088 Mixed Infection with Severe Fever with Thrombocytopenia Syndrome Virus and Two Genotypes of Scrub Typhus in a Patient, South Korea, 2017).
Do you also find mixed (or co) infection different in single patient in your study? If you find this, please also put this data in your study.

Response: We thank the Reviewer for their thoughtful review of our manuscript. Mixed or coinfection occurring in patients is not unusual. However, no such information was reported in our data. Further in-depth research is required to extend our knowledge of coinfection. The description has been added to the Discussion.

The revised description in the Discussion is as follows:
"It has been found that mixed or coinfection may be incurred in patients by evidence of different pathogens existing in tissue specimens (eschar and whole blood) assayed

by the sequencing of PCR clones [32-33]. However, no such information was reported in our data. Further in-depth research on the topic is required to extend our knowledge of coinfection.”

2. Jeju island, South Korea is close to Taiwan and Boryong and Taguchi genotypes of O. tsutsugamushi were found in a patient, Jeju Island, South Korea
(Am. J. Trop. Med. Hyg., 99(2), 2018, pp. 287–290. doi:10.4269/ajtmh.18-0088
Mixed Infection with Severe Fever with Thrombocytopenia Syndrome Virus and Two Genotypes of Scrub Typhus in a Patient, South Korea, 2017).

Authors showed that most isolates belonged to the Karp (49.9%; 272/545) genotype.
Did you also find Boryong and Taguchi genotypes in your study?
If you do not find these types, please give some opinion on the difference between Taiwan and Jeju Island, South Korea.

Response: We reviewed Table 1 in response to the Reviewer’s query and noted that TW-9 showed a ratio of 48 of 545 and 99.7% nucleotide sequence similarity to the Boryong strain (AM494475). In addition, only 1 of 545 isolates showed sequence similarity to the Kawasaki strain (M63383) listed in the TW-15 line. Additionally, the TW-15 strain was similar to the Taguchi strain (AF173038), with sequence similarity reaching 99.37%, indicating that only 1 isolate was closely related to the Taguchi genotype among the 545 isolates. This suggests that the Taguchi genotype is not a major strain spreading in Taiwan, even geographically close to South Korea. Surveillance and research efforts for scrub typhus are needed in the future.
