# Supplementary material for: Molecular epidemiology of scrub typhus in Taiwan during 2006–2016
Source: PLoS Negl Trop Dis. 2022 Apr 29;16(4):e0010369. doi: 10.1371/journal.pntd.0010369 (PMC9094550; doi:10.1371/journal.pntd.0010369)
Supplement: Attachment — Submitted filename: Response Letter.docx [file pntd.0010369.s003.docx]

Dear Editor: March. 21, 2022

We are submitting the revised manuscript (original number **PNTD-D-21-01603**) “Molecular epidemiology of scrub typhus in Taiwan during 2006-2016” by Hsiang-Fei Chen, Shih-Huan Peng, Kun-Hsien Tsai, Cheng-Fen Yang, Mei-Chun Chang, Yeou-Lin Hsueh, Chien-Ling Su, Ruo-Yu Wang, Pei-Yun Shu, Su-Lin Yang for consideration of publication in the PLOS Neglected Tropical Diseases.

We appreciate the thoughtful and constructive comments and suggestions provided by the editor and reviewers and have carefully revised the manuscript accordingly.

Please refer to the following pages for our responses to the comments from the reviewer, including the genotype diversity of *O. tsutsugamushi* and potential co-infection with SFTSV in Taiwan.

Your careful consideration is greatly appreciated.

We hereby declare that the manuscript has not been submitted or accepted for publication elsewhere. All authors have seen and agree with the contents of the manuscript and do not have commercial or other associations that might pose a conflict of interest.

Sincerely,

Su-Lin Yang and coauthors

Center for Diagnostics and Vaccine Development,

Centers for Disease Control,

Ministry of Health and Welfare, Taiwan, R.O.C.

**Method Comments**

**Reviewer #1:** Methods are acceptable

**Response:** thank you

**Reviewer #2:** YES

**Response:** thank you

**Result Comments**

**Reviewer #1:** The results are acceptable

**Response :** thank you

**Reviewer #2:** YES
**Response :** thank you

**Conclusions Comments**

**Reviewer #1:** The conclusions are acceptable

**Response :** thank you

**Reviewer #2:** YES

**Response :** thank you

**Summary and General Comments**

**Reviewer #1**: The current form of the manuscript is excellent
**Response:** We thank the Reviewer for the comments.

**Reviewer #2**: Authors studied the tsa56 genotypes of 545 O. tsutsugamushi clinical isolates throughout Taiwan, including offshore islands, from 2006 to 2016 to elucidate the molecular epidemiology of scrub typhus in Taiwan and data indicate the widespread presence of tsa56 genotypes closely related to Thailand and Korean strains and the presence of the unique endemic strains TW-12, TW-22, TW-29, and TW-36 in Taiwan.

**1.** Several reports reported mixed (or co) infection of different genotypes in a patient in SE and East Asia

1. Emerg Infect Dis. 2018 Aug;24(8):1520-1523. doi: 10.3201/eid2408.171622. Dual Genotype Orientia tsutsugamushi Infection in Patient with Rash and Eschar, Vietnam, 2016.
2. Am. J. Trop. Med. Hyg., 99(2), 2018, pp. 287–290. doi:10.4269/ajtmh.18-0088 Mixed Infection with Severe Fever with Thrombocytopenia Syndrome Virus and Two Genotypes of Scrub Typhus in a Patient, South Korea, 2017).
Do you also find mixed (or co) infection different in single patient in your study? If you find this, please also put this data in your study.

**Response:** We thank the Reviewer for their thoughtful review of our manuscript. As Reviewer’s suggestion, mixed or coinfection occurred in patients is not unusual. In fact, we are interested in this important issue. More than 2000 sera sample (suspected arthropod -borne specimens) per year, from 2016 ~ 2021, were used for regularly SFTSV screening by real-time RT PCR at our institute. So far, no SFTSV coinfection has been found. At present, only one case had been identified in 2020. This led us to suggest that SFTSV is not an endemic disease in Taiwan. In addition, among the 545 isolates we isolated, no other different genotypes have been found. Therefore, further in-depth research is required to extend our knowledge of coinfection. The description has been added to the Discussion **(line 289-294)**, and add two references **(line 452-459)** in revised manuscript**.**

***The revised description in the Discussion is as follows:***

“It has been found that mixed or coinfection may be incurred in patients by evidence of different pathogens existing in tissue specimens (eschar and whole blood) assayed by the sequencing of PCR clones [32-33]. In fact, we are interested in this important issue. However, no additional information, such as SFTSV coinfection or mixed genotypes of scrub typhus, was found in our data. Further in-depth research on the topic is required to extend our knowledge of coinfection”.

**2.** Jeju island, South Korea is close to Taiwan and Boryong and Taguchi genotypes of *O. tsutsugamushi* were found in a patient, Jeju Island, South Korea
(Am. J. Trop. Med. Hyg., 99(2), 2018, pp. 287–290. doi:10.4269/ajtmh.18-0088 Mixed Infection with Severe Fever with Thrombocytopenia Syndrome Virus and Two Genotypes of Scrub Typhus in a Patient, South Korea, 2017).

Authors showed that most isolates belonged to the Karp (49.9%; 272/545) genotype.
Did you also find Boryong and Taguchi genotypes in your study?
If you do not find these types, please give some opinion on the difference between Taiwan and Jeju Island, South Korea.

**Response:** We reviewed Table 1 in response to the Reviewer’s query and noted that TW-9 showed a ratio of 48 of 545 and 99.7% nucleotide sequence similarity to the Boryong strain (AM494475). In addition, only 1 of 545 isolates showed sequence similarity to the Kawasaki strain (M63383) listed in the TW-15 line. Additionally, the TW-15 strain was similar to the Taguchi strain (AF173038), with nucleotide sequence similarity reaching 99.37%, indicating that only 1 isolate was closely related to the Taguchi genotype among the 545 isolates. These results implicate that the Boryong and closely related TW-9 strains are prevalent in South Korea and Taiwan, respectively. In addition, our result showed that the Taguchi genotype has not yet become the main prevalent strain in Taiwan. Taiwan is geographically close to South Korea. Surveillance and research efforts for scrub typhus are needed in the future. The description has been added to the Discussion **(line 295-304)**, and add two references **(line 456-463)** in revised manuscript.

***The revised description in the Discussion is as follows:***

We noted that TW-9 showed a ratio of 48 of 545 and 99.7% nucleotide sequence similarity to the Boryong strain (AM494475). In addition, only 1 of 545 isolates showed sequence similarity to the Kawasaki strain (M63383) listed in the TW-15 line (Table 1). Additionally, the TW-15 strain was similar to the Taguchi strain (AF173038) [33], with nucleotide sequence similarity reaching 99.37%, indicating that only 1 isolate was closely related to the Taguchi genotype among the 545 isolates. These results suggest that the Boryong and closely related TW-9 strains are prevalent in South Korea [34] and Taiwan, respectively. In addition, our results showed that the Taguchi genotype has not yet become the main prevalent strain in Taiwan. Taiwan is geographically close to South Korea. Surveillance and research efforts for scrub typhus are needed in the future.

**3.** Cases were detected in the Middle East and South America and also reported on Chiloé Island in southern Chile (N Engl J Med 2016; 375:954-961)
Could you put this (also reference(s)) in line 35 and 36?

**Response:** We thank the Reviewer for the comment. The sentence at author summary has been revised as: “Scrub typhus is a mite-borne disease and the most common rickettsial disease in the regions including Asia-Pacific, Middle East, and South America. **(line 36)**

In addition, a similar description in the Discussion **(lines 329-331)** has been cited this reference listed at Reference 42 **(line 487-489)**.

4. In line 91, please write the full name of “qPCR”

**Response:** has been corrected to “real-time polymerase chain reaction (PCR)” **(line 91)**

5. In line 129, please put more information about “Zeiss” such as the name of the city and country.
**Response:** has been corrected to “(Zeiss, Axio Imager 2, Jena, Germany)” **(line 129)**

6. In line 140, please put more information about “QIAGEN” such as the name of the city and country.
**Response:** has been corrected to “(QIAGEN, Hilden, Germany)” **(line 140)**

7. In line 150, please put more information about “MEGA version 7” such as the name of the city and country.

**Response:** The website and reference have been indicated: (<http://www.megasoftware.net/>) and Reference 19 in revised manuscript. **(line 150) (line 409-411)**

8. In line 307 and 310, please write italics about “O. tsutsugamushi”.

**Response:** We thank the Reviewer for the comment. The mistake has been corrected to *O. tsutsugamushi* in the revised manuscript. **(line 309, 314)**
